# Nonclassical Effects Based on Husimi Distributions in Two Open Cavities Linked by an Optical Waveguide

**DOI:** 10.3390/e22070767

**Published:** 2020-07-13

**Authors:** Abdel-Baset A. Mohamed, Hichem Eleuch

**Affiliations:** 1 Department of Mathematics, College of Science and Humanities in Al-Aflaj, Prince Sattam bin Abdulaziz University, Al-Aflaj 710-11912, Saudi Arabia; 2 Faculty of Science, Assiut University, Assiut 71516 , Egypt; 3 Institute for Quantum Science and Engineering, Texas A&M University, College Station, TX 77843, USA; 4 Department of Applied Sciences and Mathematics, College of Arts and Sciences, Abu Dhabi University, Abu Dhabi 59911, UAE

**Keywords:** quantum coherence, Wehrl entropy, optical waveguide, quantum well

## Abstract

Nonclassical effects are investigated in a system formed by two quantum wells, each of which is inside an open cavity. The cavities are spatially separated, linked by a fiber, and filled with a linear optical medium. Based on Husimi distributions (HDs) and Wehrl entropy, we explore the effects of the physical parameters on the generation and the robustness of the mixedness and HD information in the phase space. The generated quantum coherence and the HD information depend crucially on the cavity-exciton and fiber cavity couplings as well as on the optical medium density. The HD information and purity are lost due to the dissipation. This loss may be inhibited by increasing the optical susceptibility as well as the couplings of the exciton-cavity and the fiber-cavity. These parameters control the regularity, amplitudes, and frequencies of the generated mixedness.

## 1. Introduction

Since the early eighties, cavity quantum electrodynamics (QED) experiments have been central to several prominent studies, such as atomic lifetime enhancement, controlling quantum systems, realization of Schrödinger’s cat experiment, quantum non-demolition measurement, manipulation of quantum coherence, and realization of quantum feedback schemes, to cite a few [1,2,3,4,5]. In parallel to these studies, semiconductor cavity QED systems have shown similar QED phenomena [6]. Nowadays, the atomic and semiconductor cavity QED systems [7,8,9] present several applications in quantum computing to conceive quantum gates and networks [10]. In particular, spatially separated qubits are proposed as potential physical realization for quantum networks [11,12,13,14,15,16,17].

Quantum effects, such as quantum coherence and mixedness, have been introduced as main resources for quantum computation [18], and they are equivalent only in closed systems. Mixedness, which can be quantified by different measures, such as von Neumann entropy, linear entropy and Wehrl entropy, has been explored in several systems [19,20,21,22]. Entanglement is a specific kind of quantum correlations, and has been extensively studied for many systems [20,22,23,24,25,26]. It is linked to several applications in quantum information, such as quantum processing [27], key distribution [28], teleportation [29], cryptography [30], and quantum memory [31,32].

Towards understanding the dynamics of the nonclassical effects, the Husimi quasiprobability distributions are utilized as the preferred tool to investigate the phase space information and the mixedness. Phase-space Husimi distributions (HDs) [33] are usually used to investigate quantum effects. They are always positive distributions [34], and they are crucial tools for exploring the phase-space coherence. Based on the HDs, Wehrl density and Wehrl entropy [35] are introduced to quantify the phase space information and the mixedness [36]. These HDs quantifiers were studied only to explore the phase quantum state spaces for one-qubit or one-qutrit inside a cavity [34,36,37]. HDs for multi-qubit systems still need to be investigated.

Our main motivation here is first to study the contribution of the collective qubit systems linked by an optical waveguide in order to investigate the nonclassical effects, as important resources for the quantum information processing. This is of importance to the conception of quantum networks based on distributed quantum nodes (qubits) for the storage and manipulation of quantum data [32,38,39]. Recent applications for qubit-cavity systems are based on realizing a multi-target-qubit unconventional geometric phase gate [40], entanglement stabilization protocols [41], and the network architecture [42]. We are also interested to analyze the generation and robustness of the phase space information and the mixedness in the cavity-fiber systems depending on the Husimi distribution and Wehrl entropy quantifiers. The quantum effects of these quantifiers may be an indicator to important quantum information [18]. They give sharp signatures of the irreversibility of an open system dynamics [43], the quantum phase transition and quantum coherence [44,45,46].

Therefore, the dynamics of the HD and Wehrl entropy are explored here for two nonlinear spatially separated open cavities linked by an optical waveguide. Each cavity contains one exciton quantum well and is filled by an optical linear medium.

## 2. Physical Model

Our considered system is formed by two open cavities (*A* and *B*). These cavities are linked by a waveguide and filled by a linear optical medium. Each cavity also contains a quantum well placed in a position corresponding to the maximum of the electromagnetic field [47,48,49]. We focus on the case where: (1) the driving lasers are in incident pumping; and (2) the laser, the cavities, the quantum wells, and the fiber modes have the same frequency (resonance). Additionally, we concentrate on the case of the short fiber interaction, which can be realized in usual experimental situations [39,50]. The condition of the short fiber limit is defined by $\frac{2l\bar{\nu }}{2\pi c}\ll 1$, where *l* designates the fiber length, *c* is the light speed in the fiber, and $\bar{\nu }$ is the cavity dissipation. The interaction Hamiltonian is given by:

(1) $$\begin{array}{ccc} {\hat{H}}_{int} & = & \sum_{k=A,B}{\hat{H}}_{Int-EP}^{k}+\lambda_{f}\left(\hat{f}{\hat{a}}_{k}^{†}+{\hat{f}}^{†}{\hat{a}}_{k}\right). \end{array}$$

The last two terms describe the interaction between the two exciton-photon (EP) systems and the fiber mode. $\hat{f}$ is the annihilation operator for the optical fiber mode, and $\lambda_{f}$ designates the coupling constants between the optical fiber and the microcavity modes. ${\hat{H}}_{Int-EP}^{k}$ is the interaction Hamiltonian of the *i*-EP system,

(2) $$\begin{array}{c} \;\;\;\;{\hat{H}}_{Int-EP}^{k}=\chi_{k}{\hat{a}}_{k}^{†}{\hat{a}}_{k}+\lambda_{k}\left({\hat{a}}_{k}^{+}{\hat{b}}_{k}+{\hat{b}}_{k}^{+}{\hat{a}}_{k}\right)+\varepsilon_{k}\left({\hat{a}}_{k}^{+}+{\hat{a}}_{k}\right). \end{array}$$

where ${\hat{a}}_{i}$ (${\hat{a}}_{i}^{†}$) designate the annihilation (creation) operators of the cavities. $\chi_{k}=\chi \;\left(k=A,B\right)$ present the linear optical susceptibility of the *k*-optical medium inside the cavities. ${\hat{b}}_{k}$ is the annihilation excitonic operator. The second term of the Hamiltonian represents the *k*-EP interaction with the coupling constants $\lambda_{k}\left(k=A,B\right)$. $\varepsilon_{k}$ designates the amplitudes of the *k*-pumps laser.

The master equation of the system is (we take ℏ=1) [51]:

(3) $$\begin{array}{ccccc} \frac{\partial \rho (t)}{\partial t} & = & -i\left[H_{int},\rho \left(t\right)\right] & + & \sum_{k=A,B}\kappa_{i}\left[2{\hat{a}}_{k}\rho \left(t\right){\hat{a}}_{k}^{†}-{\hat{a}}_{k}^{†}{\hat{a}}_{k}\rho \left(t\right)-\rho \left(t\right){\hat{a}}_{k}^{†}{\hat{a}}_{k}\right]\\ & & & + & \frac{\gamma_{k}}{2}\left[2{\hat{b}}_{k}\rho \left(t\right){\hat{b}}_{k}^{†}-{\hat{b}}_{k}^{†}{\hat{b}}_{k}\rho \left(t\right)-\rho \left(t\right){\hat{b}}_{k}^{†}{\hat{b}}_{k}\right]. \end{array}$$

The $\gamma_{k}$ and $\kappa_{k}$ represent the normalized excitonic spontaneous emission and the cavity dissipation rates, respectively.

For weak pumping and weak dissipations, the off-diagonal terms $2{\hat{a}}_{i}\rho {\hat{a}}_{i}^{†}$ and $2{\hat{b}}_{i}\rho {\hat{b}}_{i}^{†}$ can be neglected in Equation (3) [52,53]. Therefore, the density matrix can then be factorized, and Equation (3) becomes:

(4) $$\begin{array}{ccc} i\frac{\partial \hat{\rho }\left(t\right)}{\partial t} & = & \left\{H_{int}-i\sum_{k=A,B}\left(\kappa_{k}{\hat{a}}_{k}^{†}{\hat{a}}_{k}+\frac{1}{2}\gamma_{k}{\hat{b}}_{k}^{†}{\hat{b}}_{k}\right)\right\}\hat{\rho }\left(t\right)\\ & - & \hat{\rho }\left(t\right)\left\{H_{int}+i\sum_{k=A,B}\left(\kappa_{k}{\hat{a}}_{k}^{†}{\hat{a}}_{k}+\frac{1}{2}\gamma_{k}{\hat{b}}_{k}^{†}{\hat{b}}_{k}\right)\right\}\\ & = & {\hat{H}}_{E}\hat{\rho }\left(t\right)-{\left({\hat{H}}_{E}\;\hat{\rho }\left(t\right)\right)}^{†}, \end{array}$$

where the non-Hermitian operator $H_{E}$ has the following expression:

(5) $$\begin{array}{ccc} H_{E} & = & H_{int}-i\sum_{k=A,B}\kappa_{k}{\hat{a}}_{k}^{†}{\hat{a}}_{k}+\frac{\gamma_{k}}{2}{\hat{b}}_{k}^{†}{\hat{b}}_{k}. \end{array}$$

From Equation (4), the dynamics of the system density matrix, $\hat{\rho }\left(t\right)$, are governed by the wave function |ψ(t)〉 as:

(6) $$\begin{array}{c} \rho (t)=|\psi (t)\rangle \langle \psi (t)|. \end{array}$$

The wave function |ψ(t)〉 satisfies:

(7) $$i\frac{d}{dt}|\psi \left(t\right)\rangle =\;{\hat{H}}_{E}\;|\psi \left(t\right)\rangle ,$$

The model of Equation (1) describes two exciton-photon systems interacting with a fiber field; therefore, the total number of the excitations is identified by $\left({\hat{f}}^{†}\hat{f}+{\hat{a}}_{i}^{†}{\hat{a}}_{i}+{\hat{b}}_{i}^{†}{\hat{b}}_{i}\right)$. The Hilbert space of the wave function of the system is assumed under the the following conditions: (1) the number of excitations is restricted to 3; (2) only single photon processes are considered. Therefore, the two-exciton basis space is: $\{|\varpi_{1}\rangle ={|↓}_{A}{↓}_{B}\rangle ,|\varpi_{2}{\rangle =|↓}_{A}{↑}_{B}\rangle$, $|\varpi_{3}{\rangle =|↑}_{A}{↓}_{B}\rangle$, $|\varpi_{4}{\rangle =|↑}_{A}{↑}_{B}\rangle \}$, and the wave function |ψ(t)〉 can be represented as:

(8) $$\begin{array}{ccc} |\psi (t)\rangle & = & \sum_{l=1}^{4}|\beta_{l}\rangle ⊗|\varpi_{l}\rangle , \end{array}$$

where $|\beta_{i}\rangle =|Cav_{A},Cav_{B},Fib\rangle$ designate the cavities-fiber states, and are given by:

$$\begin{array}{ccc} |\beta_{1}\rangle & = & a_{1}|000\rangle +a_{9}|010\rangle +a_{13}|100\rangle +a_{17}|110\rangle \\ & + & a_{5}|001\rangle +a_{11}|011\rangle +x_{15}|101\rangle +a_{18}|111\rangle ,\\ |\beta_{2}\rangle & = & a_{2}|000\rangle +a_{14}|100\rangle +a_{6}|001\rangle +a_{16}|101\rangle ,\\ |\beta_{3}\rangle & = & a_{3}|000\rangle +a_{10}|010\rangle +a_{7}|001\rangle +a_{12}|011\rangle ,\\ |\beta_{4}\rangle & = & a_{4}|000\rangle +a_{8}|001\rangle . \end{array}$$

Using Equation (7), the dynamics of the time-dependent coefficients $a_{n}\left(t\right)\left(n=1-18\right)$ are governed by the following differential equations:

(9) $$\begin{array}{ccc} {\dot{a}}_{1} & = & \varepsilon_{A}a_{13}+\varepsilon_{B}a_{9},\\ {\dot{a}}_{2} & = & -i\lambda_{B}a_{9}-{\tilde{\gamma }}_{B}a_{2},\\ {\dot{a}}_{3} & = & -i\lambda_{A}a_{13}-{\tilde{\gamma }}_{A}a_{3}+\varepsilon_{A}a_{14}+\varepsilon_{B}a_{10},\\ {\dot{a}}_{4} & = & -i\lambda_{A}a_{14}-i\lambda_{B}a_{10}-\left({\tilde{\gamma }}_{A}+{\tilde{\gamma }}_{B}\right)a_{4},\\ {\dot{a}}_{5} & = & -i\lambda_{f}a_{9}-i\lambda_{f}a_{13}+\varepsilon_{A}a_{15}+\varepsilon_{B}a_{11},\\ {\dot{a}}_{6} & = & -i\lambda_{B}a_{11}-i\lambda_{f}a_{14}-{\tilde{\gamma }}_{B}a_{6},\\ {\dot{a}}_{7} & = & -i\lambda_{A}a_{15}-i\lambda_{f}x_{10}-{\tilde{\gamma }}_{A}a_{7}+\varepsilon_{A}a_{16}+\varepsilon_{B}a_{12},\\ {\dot{a}}_{8} & = & -i\lambda_{A}a_{16}-i\lambda_{B}a_{12}-\left({\tilde{\gamma }}_{A}+{\tilde{\gamma }}_{B}\right)a_{8},\\ {\dot{a}}_{9} & = & -i\lambda_{B}a_{2}-i\lambda_{f}a_{5}-\kappa_{B}a_{9}-i\chi_{B}a_{9}+\varepsilon_{A}a_{17}+\varepsilon_{B}a_{1},\\ {\dot{a}}_{10} & = & -i\lambda_{A}a_{17}-i\lambda_{B}a_{4}-i\lambda_{f}a_{7}-\left(\kappa_{B}+{\tilde{\gamma }}_{A}\right)a_{10},\\ & & \;\;-i\chi_{B}a_{10}+\varepsilon_{B}a_{3},\\ {\dot{a}}_{11} & = & -i\lambda_{B}a_{6}-i\lambda_{f}a_{17}-\kappa_{B}a_{11}-i\chi_{B}a_{11}+\varepsilon_{A}a_{18}+\varepsilon_{B}a_{5},\\ {\dot{a}}_{12} & = & -i\lambda_{A}a_{18}-i\lambda_{B}a_{8}-\left(\kappa_{B}+{\tilde{\gamma }}_{A}\right)a_{12}-i\chi^{B}a_{12}+\varepsilon_{B}a_{7},\\ {\dot{a}}_{13} & = & -i\lambda_{A}a_{3}-i\lambda_{f}a_{5}-\kappa_{A}a_{13}-i\chi^{A}a_{13}+\varepsilon_{A}a_{1}+\varepsilon_{B}a_{17},\\ {\dot{a}}_{14} & = & -i\lambda_{A}a_{4}-i\lambda_{B}a_{17}-i\lambda_{f}a_{6}-\left(\kappa_{A}+{\tilde{\gamma }}_{B}\right)a_{14},\\ & & \;\;-i\chi^{A}a_{14}+\varepsilon_{A}a_{2},\\ {\dot{a}}_{15} & = & -i\lambda_{A}a_{7}-i\lambda_{f}a_{17}-\kappa_{A}a_{15}-i\chi_{A}a_{15}+\varepsilon_{A}a_{5}+\varepsilon_{B}a_{18},\\ {\dot{a}}_{16} & = & -i\lambda_{A}a_{8}-i\lambda_{B}a_{18}-\left(\kappa_{A}+{\tilde{\gamma }}_{B}\right)a_{16}-i\chi_{A}a_{16}+\varepsilon_{A}a_{6},\\ {\dot{a}}_{17} & = & -i\lambda_{A}a_{10}-i\lambda_{B}a_{14}-i\lambda_{f}a_{15}-\left(\kappa_{A}+\kappa_{B}\right)a_{17},\\ & & \;\;\;-i\left(\chi^{A}+\chi^{B}\right)a_{17}+\varepsilon_{A}a_{9}+\varepsilon_{B}a_{13},\\ {\dot{a}}_{18} & = & -i\lambda_{A}a_{12}-i\lambda_{B}a_{16}-i\lambda_{f}a_{11}-\left(\kappa_{A}+\kappa_{B}\right)a_{18},\\ & & \;\;\;-i\left(\chi_{A}+\chi_{B}\right)a_{18}+\varepsilon_{A}a_{11}+\varepsilon_{B}a_{15}. \end{array}$$

where ${\tilde{\gamma }}_{i}=\frac{\gamma_{i}}{2}$. $\lambda_{k}$ are the coupling rates between excitons and photons, while $\varepsilon_{k}$ are the laser pump amplitudes. In the numerical calculations, we take $\gamma_{i}=\kappa_{i}=\kappa$. The phase space information and the mixture will be studied via the Husimi distributions. In the two-exciton basis states $\{|\varpi_{i}\rangle \}\left(i=1-4\right)$, the reduced density matrix of the two-exciton system is represented as:

(10) $$\begin{array}{c} {\hat{\rho }}^{E_{s}}\left(t\right)={\mathrm{Tr}}_{\mathrm{Cavities}+\mathrm{Fiber}}\{|\psi \left(t\right)\rangle \langle \psi \left(t\right)|\}. \end{array}$$

Then the reduced density matrices of the *k*-exciton (k=A,B) are defined by $\rho^{E_{1}\left(E_{2}\right)}\left(t\right)={\mathrm{Tr}}_{E_{2}\left(E_{1}\right)}\left\{\rho^{E_{s}}\left(t\right)\right\}$.

After tracing the states of the cavity and fiber fields and the *B*-exciton, we quantify the Husimi distribution and Wehrl entropy of the *A*-exciton to explore the phase space information and the mixedness of the *A*-exciton. In the numerical calculations, the time *t* represents a unitless time normalized to $\tau_{c}$, where $\tau_{c}$ is the round trip time for a photon inside the cavity. We also normalize the parameters of the system to $1/\tau_{c}$ as: $\lambda_{i}=\lambda_{i}\tau_{c}$, $\kappa =\kappa \tau_{c}$ $\epsilon =\epsilon \tau_{c}$. The the Husimi distribution and Wehrl entropy are investigated under the effects of the mentioned physical parameters after applying the time normalization for $\tau_{c}$ of the round-trip time of photons in the cavity. For our investigation, the values of these parameters were chosen so that they are close to that of a typical experiment [54,55,56]. The choice of $\lambda_{i}$ between 1 and 0.004 gives us a margin for the Rabi frequency between 20 THz and 80 GHz. The choice of the dissipations $\gamma_{i}=\kappa_{i}=0.025$ gives a lifetime of excitons of 0.34ps and a lifetime of photons of 0.17ps (equivalent to a reflection rate of the sample of 95%).

## 3. Husimi Distribution (HD)

### 3.1. Phase Space Information of the Husimi Distribution

The HD is a tool to investigate the phase space information for a spin system. Here, we take two $\frac{1}{2}$-spins (two-exciton system). The phase space information is determined by the angles θ and ϕ [37]. The loss of information means that the HD is invariant in the phase space, and thus it is an inductor of the mixture.

Using the basis: $\left\{|\varpi_{i}\rangle \right\}$, the two-exciton Bloch coherent states can be written as [57]:

(11) $$\begin{array}{ccc} {\;|\Phi \rangle }_{AB}\; & = & \cos{\tilde{\theta }}_{A}\cos{\tilde{\theta }}_{B}|\varpi_{1}\;\rangle +e^{i\phi_{B}}\cos{\tilde{\theta }}_{A}\sin{\tilde{\theta }}_{B}|\varpi_{2}\rangle \\ & + & \;e^{i\phi_{A}}\sin{\tilde{\theta }}_{A}\cos{\tilde{\theta }}_{B}|\varpi_{3}\;\rangle +e^{i(\phi_{A}+\phi_{B})}\sin{\tilde{\theta }}_{A}\sin{\tilde{\theta }}_{B}|\varpi_{4}\;\rangle . \end{array}$$

where ${\tilde{\theta }}_{i}=\frac{1}{2}\theta_{i}\left(i=A,B\right)$. Consequently, the HD of the two-exciton system $\rho^{E_{s}}\left(t\right)$ is:

(12) $$\begin{array}{ccc} H_{AB}\left(\Phi ,t\right) & = & \frac{1}{4\pi^{2}}\langle \Phi |\rho^{E_{s}}\left(t\right){|\Phi \rangle }_{AB}, \end{array}$$

and its partial HD of the *k*-exciton (say k=A) is given by:

$$\begin{array}{ccc} H_{A}\left(\theta_{A},\phi_{A},t\right) & = & \int_{0}^{\pi }\int_{0}^{2\pi }H_{AB}\left(\Phi ,t\right)\sin\theta_{B}\;d\phi_{B}\;d\theta_{B}\\ & = & \frac{1}{4\pi }\left[1+\langle {\hat{\sigma }}_{z}^{A}\rangle \cos\theta_{A}+\langle {\hat{\sigma }}_{x}^{A}\rangle \sin\theta_{A}\cos\varphi_{A}+\langle {\hat{\sigma }}_{y}^{A}\rangle \sin\theta_{A}\sin\varphi_{A}\right]\\ & = & \frac{1}{4\pi }\left[1+X\left(t\right)\right], \end{array}$$

where $\langle {\hat{\sigma }}_{i}^{A}\rangle \left(i=x,y,z\right)$ represent the the expectation values of the Pauli matrices for the *A*-exciton associated with its reduced density matrix,

$$\begin{array}{ccc} {\hat{\rho }}^{A}\left(t\right) & = & \left(\begin{array}{cc} \rho^{11}\left(t\right)+\rho^{22}\left(t\right) & \rho^{13}\left(t\right)+\rho^{24}\left(t\right)\\ \rho^{31}\left(t\right)+\rho^{42}\left(t\right) & \rho^{33}\left(t\right)+\rho^{44}\left(t\right) \end{array}\right), \end{array}$$

where $\rho^{ij}\left(t\right)=\langle \varpi_{i}|{\hat{\rho }}^{E_{s}}\left(t\right)|\varpi_{j}\rangle$ are the elements of the two-exciton state $\rho^{E_{s}}\left(t\right)$.

**Figure 1:**
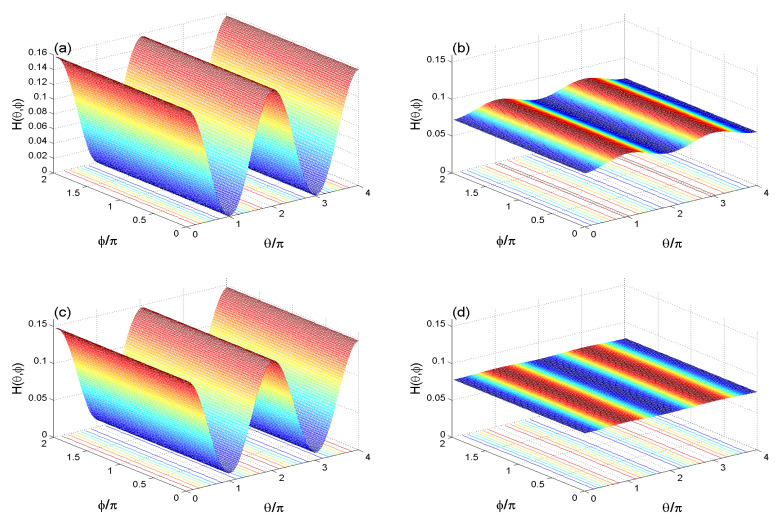
Husimi distribution H(θ,ϕ) for |110〉 and $\epsilon =10^{-3}$ for the cases $\left(\lambda_{i},\chi ,\kappa ,t_{p}\right)=\left(1,0,0,2.25\pi \right)$ in (**a**), $\left(\lambda_{i},\chi ,\kappa ,t\right)=\left(1,0,0,t_{m}\right)$ in (**b**), $\left(\lambda_{i},\chi ,\kappa ,t\right)=\left(1,2,0,t_{m}\right)$ in (**c**) and $\left(\lambda_{i},\chi ,\kappa ,t\right)=\left(1,0,0.1,t_{m}\right)$ in (**d**).

**Figure 2:**
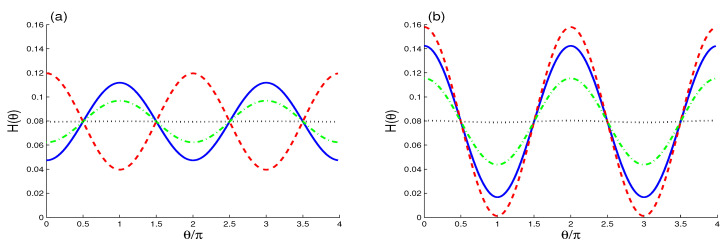
Husimi distribution H(θ) at $t=t_{m}$ for $\left(\lambda_{A},\lambda_{B},\lambda_{f}\right)=\left(1,1,0.2\right)$ in (**a**) and $\left(\lambda_{A},\lambda_{B},\lambda_{f}\right)=\left(0.2,0.2,1\right)$ in (**b**) for the cases: χ=κ=0 (solid curves), (χ,κ)=(2,0) (dashed curves), (χ,κ)=(0,0.025) (dash-dot curves) and (χ,κ)=(0,0.2) (doted curves).

Figure 1 and Figure 2 show the dependence of the HD of the *A*-exciton $H\left(\theta ,\phi \right)=H_{k}\left(\theta ,\phi \right)$ on the evolution parameter, the external environment, the cavity-exciton, and the fiber-cavity coupling rates as well as on the optical susceptibility.

**Figure 3:**
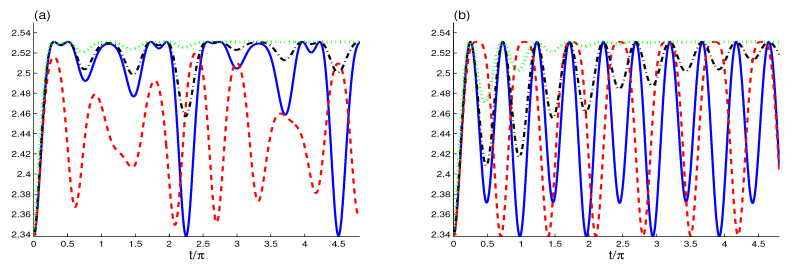
The Wehrl entropy (WE) for $\left(\lambda_{A},\lambda_{B},\epsilon \right)=\left(1,1,10^{-3}\right)$ for the different cases: χ=κ=0 (solid curves), (χ,κ)=(2,0) (dash curves), (χ,κ)=(0,0.025) (dashed-doted curves) and (χ,κ)=(0,0.1) (doted curves) for $\lambda_{f}=1$ in (**a**) and $\lambda_{f}=0.2$ in (**b**).

Figure 1 illustrates the 3D-Husimi distribution H(θ,ϕ,t) of the *A*-exciton when the total system is initially in the state |110〉 ($a_{17}=1$) where θ∈[0,4π] and ϕ∈[0,2π]. The values of the evolution parameter *t* were chosen based on the results of the Wehrl entropy mixedness. The *A*-exciton is in a pure state at $t_{p}=0,2.25\pi ,...$, and it is in a mixed state at several times, one of them being $t_{m}=2.6101\pi$ (see Figure 3a). To show the effect of the cavity-exciton interaction on the initial Husimi distribution $H(\theta ,\phi ,t_{p})$, the Husimi distribution is displayed in Figure 1a. The Husimi distribution of the initial *A*-exciton state $\rho^{A}\left(0\right)=|1_{A}\rangle \langle 1_{A}|$ has a regular oscillatory behavior. We note that the 3D-Husimi distribution H(θ,ϕ) depends only on the angle θ, and that it has a regular 2π-oscillatory surface. The maxima of the HD are at θ=2nπ,(n=1,2,…) whereas its minima are at θ=(2n+1)π, (n=0,1,2,…). In this case, the *A*-exciton HD oscillate between its extreme values $\frac{1+\cos\theta }{4\pi }$. For θ=0 we have $H_{\min}=0$, and for θ=π we get $H_{\max}=\frac{1}{2\pi }≃0.16$. The counter-plot of the HD appears as color lines in the θ−ϕ plane, while parallel lines of the ϕ-axis illustrate the dependence of the maxima and minima of the HD on the phase space angular variables.

Figure 1b, at $t_{m}=2.6101\pi$, shows the effect of the [cavity-exciton]-fiber-[cavity-exciton] interactions, with the strong couplings $\lambda_{A}=\lambda_{B}=\lambda_{f}=1$, on the *A*-exciton HD that leads to a decreasing of the amplitudes of the HD, where the maxima and minima of the HD tend to the stationary HD surface $H\left(\theta ,\phi \right)=\frac{1}{2}H_{\max}≃0.08$ (that appears clear with a large value of damping). The dependence of the HD on the θ-angle can be controlled by the evolution parameter *t*.

Figure 1c, shows the Husimi distribution for an optical susceptibility χ=2 with the same taken strong interaction couplings in Figure 1a. In this case, we observe that the oscillation frequency of HD, due to the unitary interactions, is at the same values as the case χ=0. While the oscillation amplitudes of HD increase. The linear optical susceptibility leads to an increase of the dependence of the HD on the θ-angle, and the maxima and minima of the HD return to their initial values approximately. We deduce that when the cavities are filled by a linear optical medium, the phase space information is more robust against the interactions.

Figure 1d shows the effect of the dissipation cavities on the maxima and minima of HD for the case where the same strong interaction couplings taken in Figure 1a. The amplitudes of the HD oscillations are affected, where the maxima and minima of HD remain at the same stationary value. The HD oscillations vanish after incasing the dissipations. The increase of the dissipation leads to a stationary HD surface of a 0.8 value (see Figure 1d); therefore, it leads to an erasing of the phase space information.

Based on the property that HD depends only on the angle θ, we can use the partial 2D-Husimi distribution H(θ)=H(θ,π) for the fixed value ϕ=π to analyze the phase space information under the effects of the external environment, the cavity-exciton, and the fiber-cavity coupling rates as well as the optical susceptibility; see Figure 2.

In Figure 2a, we display the dependence of the 2D-Husimi distribution H(θ) on the angle θ when the coupling strength of the fiber-cavity interaction is reduced to $\lambda_{f}=0.2$. We observe that the 2D-Husimi distribution H(θ) has regular oscillations with a 2π-period around the stationary value H(∞)=0.08. For the case χ=0 and $\lambda_{f}=0.2$ and the maxima of the HD are at θ=(2n+1)π, (n=0,1,2,…) whereas its minima are at θ=2nπ,(n=1,2,…); this is an opposite behavior to the case where the strong fiber-cavity interaction coupling $\lambda_{f}=1$.

The dashed curve shows the effect of the optical susceptibility χ=2 for the case $\lambda_{f}=0.2$. We note the same observations of the case $\lambda_{f}=1$. Dash-dot and doted curves show the effect of the cavity dissipation. The observations of the effects of the external environment and the optical susceptibility, for $\lambda_{f}=1$, are confirmed for $\lambda_{f}=0.2$.

Figure 2b shows the sensitivity of the HD oscillations to the exciton-cavity coupling constants. If they are reduced to $\lambda_{A}=\lambda_{B}=0.2$, the amplitudes of the HD oscillations present notable changes. By comparing the cases $\left(\lambda_{A},\lambda_{B},\lambda_{f}\right)=\left(0.2,0.2,1\right)$ and $\left(\lambda_{A},\lambda_{B},\lambda_{f}\right)=\left(1,1,0.2\right)$, we find that the amplitudes of the HD oscillations increase due to enhancement of the optical susceptibility. We find that the weak exciton-cavity couplings lead to an increasing of the dependence of the HD on the θ-angle, and the phase space information is more robust against the interactions.

Finally, we can deduce that the HD oscillations may be controlled by the exciton-cavity and fiber-cavity coupling constants as well as by the susceptibility of the optical medium.

### 3.2. Wehrl Entropy

One of the most important applications of the Husimi distribution in quantum information is the Wehrl entropy (WE) [35]. It is used to quantify the mixture (purity loss/mixedness) for open systems in the phase space determined by the angels θ∈[0,π] and ϕ∈[0,2π] [58]. The partial Wehrl density of the *k*-exciton $D_{k}\left(\theta_{k},\phi_{k},t\right)$, is defined by:

(13) $$\begin{array}{c} D_{k}\left(\theta_{k},\phi_{k},t\right)=-H_{k}\left(\theta_{k},\phi_{k},t\right)\;\ln\left[H_{k}\left(\theta_{k},\phi_{k},t\right)\right]. \end{array}$$

from this expression we can deduce the partial WEs of the *A*-exciton as:

(14) $$\begin{array}{ccc} S(t) & = & \int_{0}^{2\pi }\int_{0}^{\pi }D_{A}\left(\theta_{A},\phi_{A},t\right)\;\sin\theta_{A}\;d\theta_{A}\;d\phi_{A}\\ & = & \ln4\pi -\frac{1}{4\pi }\int_{0}^{2\pi }\int_{0}^{\pi }\left[1+X\left(t\right)\right]\;\ln\left[1+X\left(t\right)\right]\;\sin\theta_{A}\;d\theta_{A}\;d\phi_{A}. \end{array}$$

If the two excitons start in the lower state ${|↓}_{A}{↓}_{B}\rangle$, then the initial value of the partial WE is calculated as [57]:

(15) $$\begin{array}{ccc} S(0) & = & -2\int_{0}^{\frac{1}{2}\pi }\;\;\;\;\;\;\sin2\theta \cos^{2}\theta \ln\left[\cos^{2}\left(\theta \right)/2\pi \right]\;d\theta =2.34. \end{array}$$

The WE function S(t) [57] verifies,

(16) $$\begin{array}{c} 2.3379\leq S(t)\leq \ln(4\pi ). \end{array}$$

The initial value of S(0) is for a pure state. The maximal value $S^{\max}=\ln\left(4\pi \right)$ indicates that the state is totally mixed. The Wehrl entropy is a good quantifier that provides important information about the transition processes (mixedness) between a pure state and a steady mixed state. In the real quantum devices, the decoherence is unavoidable, which destroys the pure state. In other words, the Wehrl entropy mixedness is the measure of the environment effect on the system.

For the case of one-qubit systems, there is a relation between the Wehrl entropy and the other entropies as von Neumann entropy and linear entropy [59]. It was proven that they exhibit similar behaviors.

In Figure 3, the WE function S(t) is plotted for different values of $\lambda_{i}\left(A,B,f\right)$, χ and κ. The solid curve of Figure 3 shows the effect of the strong [cavity-exciton]-fiber-[cavity-exciton] interaction couplings $\lambda_{A}=\lambda_{B}=\lambda_{f}=1$ in the absence of the optical medium and the dissipation. The Wehrl entropy S(t) shows that the interactions lead to a generation of the mixedness of the *A*-exciton with a quasi-regular oscillatory behavior. In each period, the Wehrl entropy reaches its maximum value $S^{\max}\left(t\right)=\ln\left(4\pi \right)$, and remains there for a short interval. In these stability intervals, the *A*-exciton is in a maximally mixed state. The generated maximally mixed state as an initial state can be useful to realize quantum information processes. The Wehrl entropy returns instantaneously to its initial value at the end of each period (this means that the *A*-exciton returns to its initial pure state $|1_{A}\rangle$).

The dashed curve of Figure 3a shows how the optical susceptibility affects the dynamical behavior of the generated *A*-exciton mixedness. The amplitudes and the period of the oscillations of the Wehrl entropy are decreased by an increasing of the optical susceptibility, i.e., the generated mixedness is reduced due to the increase of the optical susceptibility.

The dash-dot and doted curves show the effect of the external environment on the generated *A*-exciton mixedness. The increase of the cavity dissipations leads to the increase of the minima of the Wehrl entropy. The WE function S(t) reaches stationary behavior quickly, i.e., it leads to an acceleration of the *A*-exciton, causing it to be in a steady mixed state for a long time. The generated maximally mixed state can be used as an initial state to realize quantum computing processes [60,61] and qubit-channel metrology [62].

The effect of the strength of the fiber-cavity interaction on the generated mixedness is shown in Figure 3b. The Wehrl entropy is displayed when the coupling strength of the fiber-cavity interaction is reduced to $\lambda_{f}=0.2$. We observe that the WE function S(t) has a regular oscillatory behavior with a π period. The period, the amplitude, and the stability intervals of the generated mixedness are less than the case where $\lambda_{f}=1$. The dashed curve of Figure 3b shows that the Wehrl entropy is improved by increasing the optical susceptibility, unlike the one in the case of the strong fiber-cavity interaction coupling. In the absence of the dissipation (see the dash-dot and doted curves of Figure 3b), we note that the decrease of the fiber-cavity coupling leads to delaying the WE from reaching the stationary value S(∞)≈ln(4π) and the steady mixed state.

Our physical interpretations of the HD-information and the Wehrl entropy-mixedness in qubit-cavity systems linked by a waveguide mode contribute to updating more potential applications for distributed quantum information processing that are based on realizing a multi-target-qubit unconventional geometric phase gate in a multi-cavity system [40], as well as entanglement stabilization protocols between two superconducting qubits that are coupled to distant cavities [41] and the architecture of the cavity-based quantum network [42]. It is found that certain coupling parameters of the exciton-cavity and the fiber-cavity interactions control the effects of the optical linear medium and the environment, which are major obstacles to the applications of the quantum information technology.

## 4. Conclusions

In this investigation, we have studied the Husimi distributions and Wehrl entropy in a system formed by two open cavities linked by a waveguide. Each cavity contains one exciton and is filled by an optical linear medium. Based on Husimi distributions and Wehrl entropy, we have explored the effects of the optical susceptibility, the cavity dissipation, the coupling of the exciton-cavity and the fiber-cavity interactions. These parameters lead to notable changes in the dynamical behavior of the HD information and the generated mixedness. The generated HD information and the qubit purity are lost completely due to the excitonic spontaneous emission and the cavity dissipations. The mixedness and the HD information may be inhibited by increasing the optical susceptibility and the couplings of the exciton-cavity and the fiber-cavity. Therefore, we can deduce that the HD-information and mixedness depend crucially on the cavity-exciton and fiber cavity interactions as well as on the optical medium density. These physical parameters control the regularity, amplitudes, and frequencies of the generated mixedness. Since the studied system is very compact and can be realized experimentally, we expect that it would be very useful in the design of quantum components involved in quantum information processes.
